# Sulodexide reduces glucose induced senescence in human retinal endothelial cells

**DOI:** 10.1038/s41598-021-90987-w

**Published:** 2021-06-01

**Authors:** A. Gericke, K. Suminska-Jasińska, A. Bręborowicz

**Affiliations:** 1 Department of Pathophysiology, Poznan University of Medical Sciences, Ul. Rokietnicka 8, 60-806 Poznan, Poland; 2 Department of Ophthalmology, University Medical Center, Johannes Gutenberg University Mainz, Mainz, Germany

**Keywords:** Cell biology, Medical research

## Abstract

Chronic exposure of retinal endothelium cells to hyperglycemia is the leading cause of diabetic retinopathy. We evaluated the effect of high glucose concentration on senescence in human retinal endothelial cells (HREC) and modulation of that effect by Sulodexide. Experiments were performed on HREC undergoing in vitro replicative senescence in standard medium or medium supplemented with glucose 20 mmol/L (GLU) or mannitol 20 mnol/L (MAN). Effect of Sulodexide 0.5 LRU/mL (SUL) on the process of HREC senescence was studied. Glucose 20 mmol/L accelerates senescence of HREC: population doubling time (+ 58%, p < 0.001) β-galactosidase activity (+ 60%, p < 0.002) intracellular oxidative stress (+ 65%, p < 0.01), expression of p53 gene (+ 118%, p < 0.001). Senescent HREC had also reduced transendothelial electrical resistance (TEER) (− 30%, p < 0.001). Mannitol 20 mmol/L used in the same scenario as glucose did not induce HREC senescence. In HREC exposed to GLU and SUL, the senescent changes were smaller. HREC, which became senescent in the presence of GLU, demonstrated higher expression of genes regulating the synthesis of Il6 and VEGF-A, which was reflected by increased secretion of these cytokines (IL6 + 125%, p < 0.001 vs control and VEGF-A + 124% p < 0.001 vs control). These effects were smaller in the presence of SUL, and additionally, an increase of TEER in the senescent HREC was observed. Chronic exposure of HREC to high glucose concentration in medium accelerates their senescence, and that process is reduced when the cells are simultaneously exposed to Sulodexide. Additionally, Sulodexide decreases the secretion of IL6 and VEGF-A from senescent HREC and increases their TEER.

## Introduction

Diabetes mellitus is nowadays a common disease, causing dysfunction of the vascular endothelial cells, which results in various pathologies within the microvascular and macrovascular system^1^. Diabetic retinopathy is one of the most common consequences of diabetes mellitus and a leading cause of visual impairment in that group of patients^2^. In the early stages of the disease, it is asymptomatic, but at that time, changes in the endothelial cells have already started. They include the abnormal secretory activity of the cells, reduction of the endothelial glycocalyx thickness, disruption of the blood-retinal barrier, thickening of the basement membrane and degeneration of capillaries. Later, leakage and proliferation of the retinal microvessels due to progressive ischemia lead to diabetic macular oedema and bleeding into the retina and vitreous body^3^.

Hyperglycemia, which is characteristic for diabetic patients, causes endothelial dysfunction via different mechanisms, such as damage to the glycocalyx^4^, oxidative stress^5^, activation of the inflammation, induction of imbalance between coagulation and fibrinolysis, and stimulation of cellular senescence^6^. High glucose-dependent hyperosmolality may also contribute to the promotion of angiogenesis in retinal endothelial cells and retinopathy^7^. The senescent phenotype of retinal endothelial cells may contribute to abnormal angiogenesis in retinopathy^8^. High glucose level, as well as advanced glycation end products, accelerate thesenescence of endothelial cells^9,10^. Therefore, inhibition of retinal endothelial senescence may be an effective way of slowing the progress of diabetic retinopathy^11^. Previously, we found that senescence of human venous and arterial endothelial cells was slowed down by Sulodexide, which is a mixture of glycosaminoglycans: low molecular-weight heparin and dermatan sulphate^12,13^. Sulodexide also reduced glucose toxicity towards human venous endothelial cells^14^.

In the presented study, we tested the hypothesis that senescence of retinal endothelial cells caused by chronic exposure to high glucose concentration is slowed down by Sulodexide. We also evaluated the effect of Sulodexide on senescent retinal endothelial cells.

## Material and methods

Experiments were performed on HREC (#HEC09, Neuromics, Edina, MN, USA) in in vitro culture. Cells were seeded in 75 cm^2^ culture flasks coated with AlphaBioCoat Solution (#AC00, Neuromics, Edina, MN, USA) and were grown in Endo Growth Medium (EKG001, Neuromics, Edina, MN, USA) supplemented with fetal bovine serum 0.5% until they formed monolayers. Then the cells were harvested with cell detachment solution (#ADF001, Neuromics, Edina, MN, USA) and seeded in quadruplicates into 25 cm^2^ coated, as described above, culture flasks at a density of 1.5 × 10^5^ cells/flask.

We studied the senescence of HREC in two sets of experiments. In the first one, we compared the effects of mannitol and glucose, used at the same concentrations of the HREC senescence. The following experimental groups were studied:

- *Control group* exposed to the standard culture medium (glucose concentration 5.0 mmol/L).
- *Glucose group* exposed to standard medium supplemented with glucose 20 mmol/L.
- *Mannitol group* exposed to standard medium supplemented with mannitol 20 mmol/L.

In the second set of the experiments, we studied the effect of Sulodexide on the glucose-induced senescence of HREC. The following experimental groups were established:

- *Control group* exposed to the standard culture medium (glucose concentration 5.0 mmol/L).
- *Glucose group* exposed to standard medium supplemented with glucose 20 mmol/L.
- *Glucose-Sulodexide group* exposed to standard medium supplemented with glucose 20 mmol/L and Sulodexide 0.5 LRU/mL.

Replicative senescence of the endothelial cells was induced according to the protocol established in our previous study^12^. Every four days, growing cells were harvested from the culture flasks, counted in a hemocytometer, and afterwards reseeded into 25 cm^2^ flasks at a density of 1.5 × 10^5^ cells/flask. During the experiment, ten passages were performed. Population doubling time (PDT) of the cultured cells was calculated according to the standard formula:

$$ {\text{PDT}} = \ln 2/\left[ {\ln \;\;\left( {{\text{N}}/{\text{No}}} \right)/{\text{t}}} \right] $$

where N—number of cells harvested from the flask, No—number of cells seeded into the flask, t—a time of culture.

After the last passage, cells were seeded into six wells, 24-well culture plates and into Labtec plates, which were grown to monolayers, for further study. The following functional parameters of the endothelial cells from each experimental group were studied:

*Intracellular generation of free radicals* measured with a 2′7'-dichlorodihydrofluorescein probe, which in the presence of oxidative stress is converted to fluorescent 2′7'-dichlorodihydrofluorescein. Fluorescence was measured in a microplate fluorescent reader, with excitation at 485 nm and emission at 530 nm. Generation of free radicals was expressed per amount of the cells' protein, measured in the cellular lysates with a BCA protein assay kit (# ab207002, Abcam, Cambridge, UK).

*Secretory activity of the cells* evaluated during 24 h’ incubation of the cell monolayers in the media appropriate for each group. We measured Interleukin – 6 (IL6) and Vascular Endothelial Growth Factor-A (VEGF-A) concentration in supernatants collected at the end of the incubation with standard ELISA kits (R&D Sytems, Minneapolis, MN, USA). The concentration of these cytokines was expressed per number of cells in each well, which were counted in a hemocytometer.

*Expression of genes in the endothelial cells* evaluated at the start of the experiment and after ten passages of the cells in different media as described above. In each group, cells were grown to monolayers in 6-well plates. At the end of 24 h’ exposure to the medium appropriate for each group, total RNA was isolated using the ReliaPrepTM RNA Cell Miniprep System (Promega, Madison, WI, USA) treated with DNase I using DNA-free DNase Treatment and Removal Reagent (Thermo Fisher Scientific, Waltham MA, USA). RNA was reverse-transcribed to cDNA using the Transcriptor First Strand cDNA Synthesis Kit (Roche, Basel, Switzerland). Relative levels of the mRNA of 3 genes (p 53, IL6, VEGF-A) were studied in triplicates from each experiment:

- **p53** [F:TTGCAATAGGTGTGCGTCAG; R:TCCCCACAACAAAACACCAG]
- **IL6** [F:ATGAACTCCTTCTCCACAAGC; R:GTTTTCTGCCAGTGCCTCTTTG]
- **VEGF-A** [F: CTTGCCTTGCTGCTCTACCT; R:GCAGTAGCTGCGCTGATAGA]

and normalized to the level of internal house-keeping genes:

- **Glyceraldehyde-3-Phosphate dehydrogenase** [F:TTCGTCATGGGTGTGAACC; R:GATGATGTTCTGGAGAGCCC]
- **Hypoxanthine phosphoribosyltranferase 1** [F:TGCTCGAGATGTGATGAAGG; R:TCCCCTGTTGACTGGTCATT]

Relative gene expression was calculated using the 2-∆∆Ct method^15^.

*The β-galactosidase activity* measured in the cell monolayers grown in Labtec wells with a Senescence Detection Kit (#ab6535, abcam, Cambridge, UK). Evaluation of β-galactosidase activity in the cell monolayers was studied at the beginning of the study and after ten passages in the different media. Results are expressed as % of change vs the beginning of the study.

*Transendothelial electrical resistance (TEER)* was measured using a Millicell-ERS (Merck, Poland). HREC were grown on the collagen-treated Transwell inserts, and resistance of the endothelial monolayers was measured 72 h since the cellular monolayer was established. Values of TEER are expressed as Ω x cm^2^.

In a separate set of experiments, we evaluated the effect of Sulodexide on the secretory activity of REC made senescent in control medium or in medium supplemented with glucose 20 mmol/L. After ten passages, the secretory activity of RES was studied during 24 h’ incubation in the culture medium appropriate for each group ± Sulodexide 0.5 LRU/mL. The concentration of IL6 and VEGF in the supernatant of the cells was measured with ELISA as described above, and recalculated per number of cells in each well. TEER was measured in the senescent cells after 24- hour exposure to Sulodexide 0.5 LRU/mL in the medium.

### Statistical analysis

Results are presented as mean ± SD. Analysis of the data was performed with a one-way analysis of variance. A p-value of less than 0.05 was considered significant.

## Results

**Figure 1:**
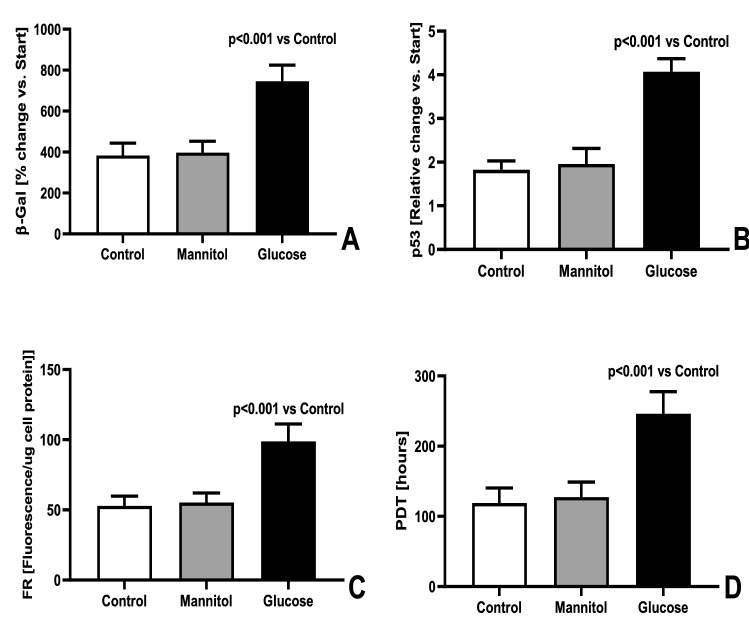
Activity of β-galactosidase (**A**), expression of p53 gene (**B**), intracellular oxidative stress (**C**) and Population doubling time (**D**) in HREC at the end of the replicative senescence in control medium (Control), medium supplemented with Mannitol 20 mmol/L (Mannitol) or medium supplemented with glucose 20 mmol/L (Glucose) (n = 8) (ANOVA analysis).

Repeated eight passages of HREC resulted in senescence of these cells, which was reflected in the control group by the increased intracellular generation of free radicals (+ 50%, p < 0.01, vs start), the elevation of SA-β-Galactosidase activity (+ 479%, p < 0.001 vs start) and prolongation of population doubling time (+ 125%, p < 0.001 vs start). Replicative senescence of HREC was accelerated in the presence of glucose 20 mmol/L, but not mannitol 20 mmol/L, added to the culture medium, as reflected by greater changes of the studied parameters (Fig. 1).

**Figure 2:**
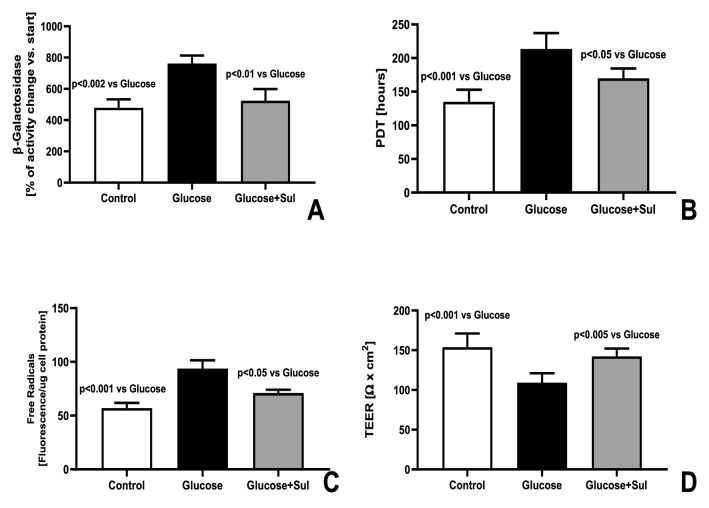
Activity of β-galactosidase (**A**), population doubling time (**B**), intracellular oxidative stress (**C**) and TEER (**D**) in HREC at the end of the replicative senescence in control medium (Control), medium supplemented with glucose 20 mmol/L Glucose) or medium supplemented with glucose 20 mmol/L and Sulodexide 0.5 LRU/mL (Glucose + Sul) (n = 8) (ANOVA analysis).

Glucose-induced senescence as reflected by increased oxidative stress, SA-β-galactosidase activity and prolongation of PDT was reduced in the HREC simultaneously exposed to Sulodexide in medium (0.5 LRU/mL), by 24%, p < 0.05, 32% p < 0.01, − 20%, p < 0.05, respectively (Fig. 2). Chronic exposure to high glucose concentration in the medium resulted in a stronger decrease of TEER, as compared to the control group, and that effect was partially modified by simultaneous treatment with Sulodexide (0.5 LRU/mL) (Fig. 2D).

**Figure 3:**
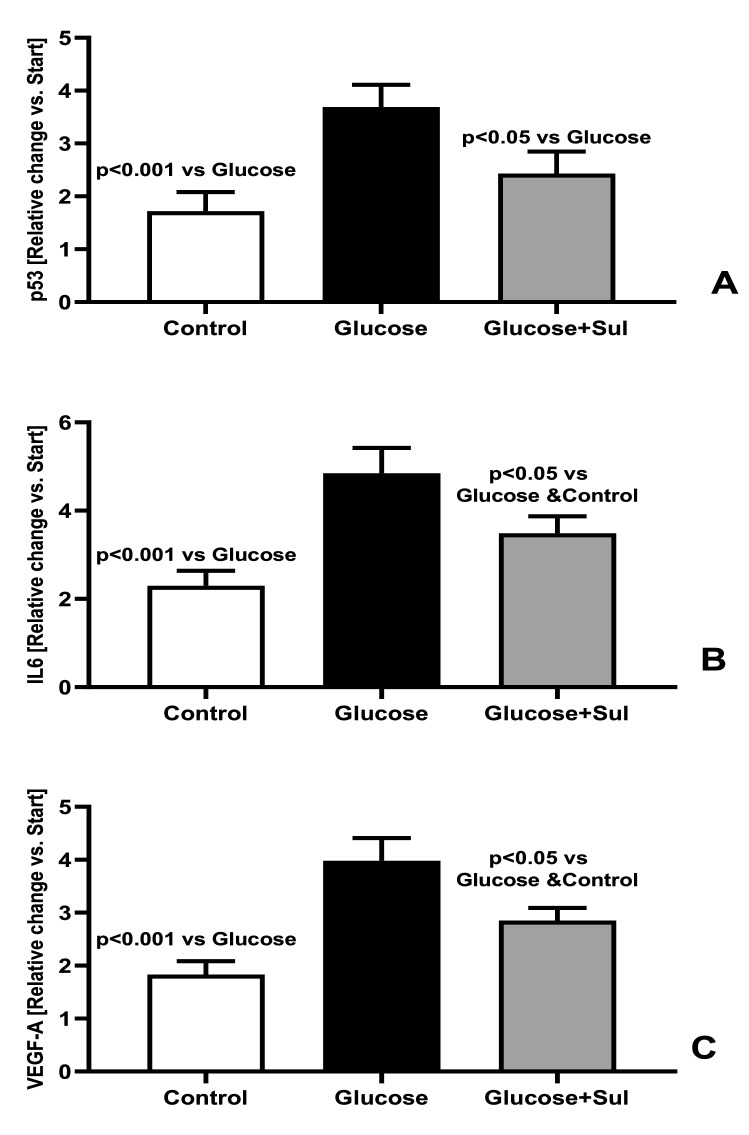
Expression of genes for p53 (**A**), IL6 (**B**) and VEGF-A (**C**), presented as change versus start of the experiment, in HREC at the end of the replicative senescence in control medium (Control) medium supplemented with glucose 20 mmo/l (Glucose) and medium supplemented with glucose 20 mmol/L and Sulodexide 0.5 LRU/mL (Glucose + Sul) (n = 8) (ANOVA analysis).

**Figure 4:**
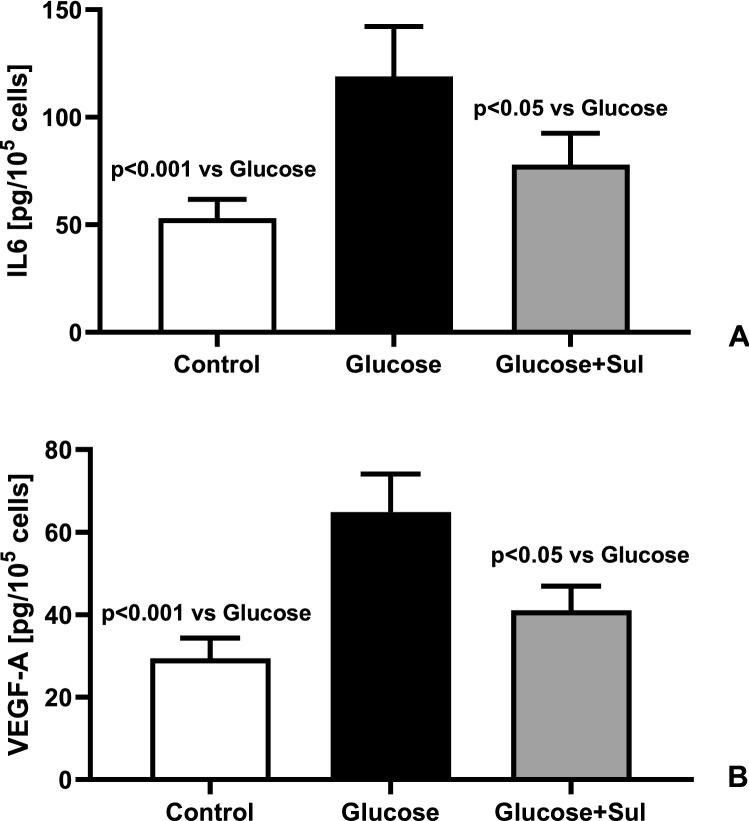
Secretion of IL6 (**A**) and VEGF-A (**B**) in HREC at the end of the replicative senescence in control medium (Control) medium supplemented with glucose 20 mmo/L (Glucose) and medium supplemented with glucose 20 mmol/L and Sulodexide 0.5 LRU/mL (Glucose + Sul) (n = 8) (ANOVA analysis).

In HREC undergoing replicative senescence, we observed increased expression of genes regulating the synthesis of p53, IL6 and VEGF-A. That process was enhanced in the presence of glucose 20 mmol/L (+ 117%, p < 0.001, + 113% p < 0.001, + 122% p < 0.001, respectively) (Fig. 3).The presence of Sulodexide in the medium partially prevented the glucose-induced increased expression of p53, IL6 and VEGF genes: − 35%, p < 0.05, − 29% p < 0.05 and − 27% p < 0.05, respectively (Fig. 3). HREC made senescent in medium supplemented with glucose secreted more IL6 (+ 125%, p < 0.001) and VEGF (+ 124%, p < 0.001) than cells made senescent in control medium (Fig. 4). The presence of Sulodexide in high glucose medium reduced the intensity of their senescence as reflected by the reduced synthesis of IL6 (− 34%, p < 0.05) and VEGF (− 27%, p < 0.05) (Fig. 4).

**Figure 5:**
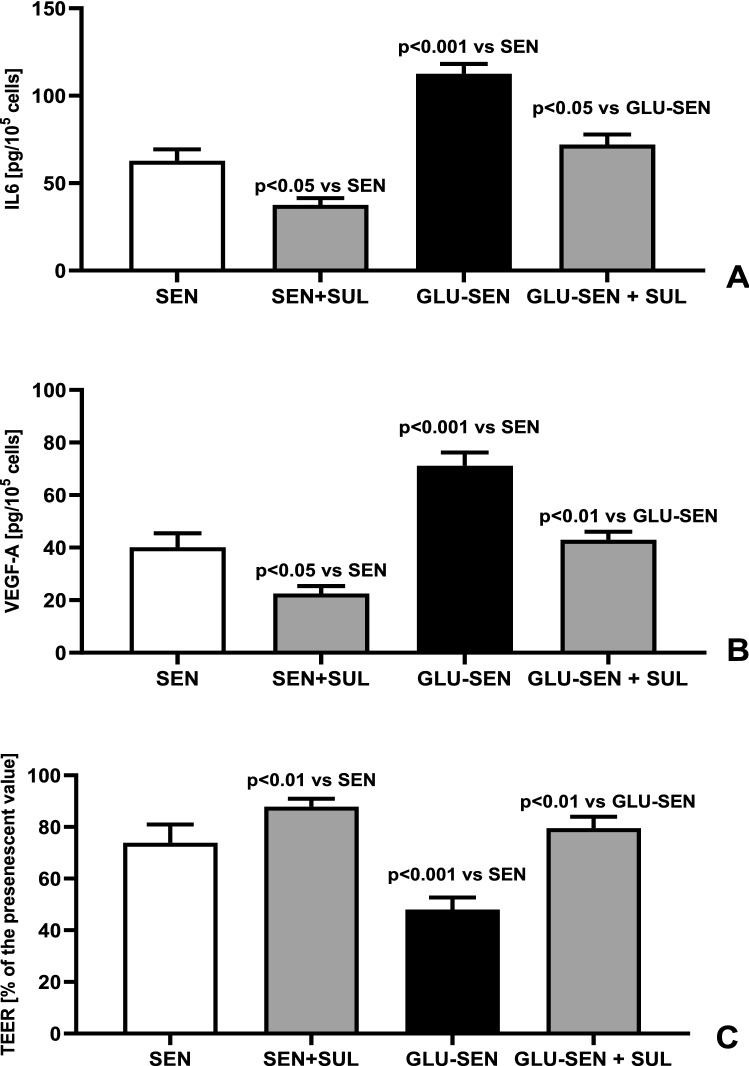
Effect of Sulodexide on synthesis of IL6 (**A**), VEGF (**B**) and value of TEER (**C**) in HREC made senescent in control medium (SEN)and in HREC made senescent in control medium + glucose 20 mmol/L (GLU-SEN) (n = 10) (ANOVA analysis).

The anti-inflammatory effect of Sulodexide was also observed in the cells which were previously made senescent in the standard medium or in medium supplemented with glucose 20 mmol/L. The secretory activity of these cells for IL6 and VEGF-A was reduced after acute exposure to Sulodexide 0.5 LRU/mL by 28%, p < 0.05 and by 22%, p < 0.05 in cells made senescent in control medium and by 23%, p < 0.05 and by 16%, p < 0.05, respectively, in cells made senescent in the presence of high glucose concentration in the medium (Fig. 5). Additionally, treatment with Sulodexide 0.5 LRU/mL caused an increase of TEER by 19%, p < 0.01 in cells made senescent in control medium and by 67%, p < 0.01 in the cells made senescent due to chronic exposure to glucose (Fig. 5C).

## Discussion

Dysfunction of retinal endothelial cells is the main pathomechanism leading to the development of diabetic retinopathy. Our results show that chronic exposure of retinal endothelial cells to high glucose concentration causes the senescence of these cells. Intracellular oxidative stress observed in retinal endothelial cells treated with glucose may be an important factor accelerating their senescence^16^. Increased β-galactosidase activity and expression of the senescent protein p53 confirmed that cells became senescent^17^. We also observed increased secretion of IL6 and VEGF, which are characteristic of diabetic retinopathy^18,19^. In our previous studies on peritoneal mesothelial cells, we found that glucose-induced oxidative stress, but not hyperosmolality, changes the properties of these cells during four weeks of culture in a medium with increased glucose concentration^20^. In retinal endothelial cells, glucose-induced oxidative stress, but not hyperosmolality, causes cell death^21^. Results from our study prove that hyperosmolality caused by the addition of glucose to the culture medium was not the factor causing HREC senescence (Fig. 1).

Sulodexide is a potential drug in the treatment of this pathology. In a clinical 12-month study, Sulodexide was shown to slow the progression of non-proliferative diabetic retinopathy^22^. The presented results confirm the strong anti-senescent and anti-inflammatory effect of sulodexide in microvascular retinal endothelial cells, which was shown previously in venous^12,23^ and arterial^13^ endothelial cells. The senescent phenotype of retinal endothelial cells predisposes patients to abnormal, excessive growth of retinal microvessels and pathological neovascularization, leading to vitreous haemorrhages^8^.

In the pathomechanisms of diabetic retinopathy, there are two possible therapeutic approaches. The first one is the direct inhibition of the angiogenic and inflammatory effects of retinal endothelial cells modified by chronic hyperglycemia^24^. Another possibility is the prevention of the development of pathologic, senescent changes in retinal endothelial cells which are responsible for their angiogenic and inflammatory action^25^. Inhibition of arginase 1 protects against premature senescence of retinal endothelial cells^11^.

Our results suggest that Sulodexide can reduce the intensity of diabetic retinopathy through both above-mentioned mechanisms. It slows the senescence of retinal endothelial cells chronically exposed to hyperglycemia (Figs. 2, 3, 4). Reduced expression of the p53 gene by 35% in cells exposed to high glucose load and Sulodexide (Fig. 3A) confirms the anti- senescent action of that drug in the presence of hyperglycemia. Sulodexide also inhibits the angiogenic and inflammatory phenotype of retinal endothelial cells made senescent due to repeated passages in the control medium or in the presence of hyperglycemia (Fig. 5). Previously, Giurdanella et al. showed that Sulodexide prevents glucose toxicity in retinal endothelial cells due to inhibition of NFκB activity, which confirms its anti-inflammatory action, which was also observed in the present study^26^.

The beneficial effect of Sulodexide in patients with diabetic retinopathy could also be due to its other biological effects. In conditions of diabetes mellitus thickness of the glycocalyx covering the surface of endothelial cells lining the retinal vessels is decreased^27^. Such changes may result in abnormal interactions between the circulating cells in the bloodstream, and increased permeability of the vascular wall, predisposing patients to the formation of haemorrhages^28^. Damage to the glycocalyx is reflected by the reduced value of TEER and results in the increased permeability of the vascular wall. Our results show that during senescence, TEER decreases and that effect is stronger when senescence is induced by chronic exposure to high glucose concentration (Fig. 2). Sulodexide partially prevents that change during senescence of HREC (Fig. 2) and partially restores TEER when applied to the senescent HREC (Fig. 5). Results from previous studies show that the decreased thickness of the endothelial glycocalyx caused by mechanical stress in the arterial vessels^29^ or during diabetes mellitus in the retinal vessels^30^ can be restored with Sulodexide. Our results suggest that restoration of the glycocalyx translates into the increased TEER, which may increase the tightness of the vascular wall.

We conclude that Sulodexide may have a beneficial effect in cases of diabetic retinopathy. It slows down hyperglycemia-dependent senescence of endothelial cells, which translates into the lower angiogenic and inflammatory impact of these cells. An important observation was that Sulodexide also has effective antiangiogenic and anti-inflammatory effects in the senescent HREC. That means that Sulodexide may be effective in retinal endothelial cells, which are already senescent. Further studies are required to explain the potential effect of Sulodexide on the endothelial glycocalyx structure and permeability of the retinal endothelial layer composed of the senescent cells to molecules with various size and electrical charge.
